# Fracture of the Patella

**Published:** 1898-07-23

**Authors:** 


### FRACTURE OF THE PATELLA.

All England has been distressed to hear of the accident which has befallen the Prince of Wales, and on every hand satisfaction has been felt at the reassuring bulletins which have been issued as to the satisfactory progress of the illustrious patient. It is idle, however, to shut our eyes to the fact that however simple an affair fracture of the patella may appear at the time, it is in many cases a matter of considerable gravity, partly in consequence of the serious interference with the utility of the limb which sometimes succeeds it, and partly from the fact that the forms of treatment by which these ulterior results can be best avoided are in themselves by no means absolutely devoid of danger.

The consequences of a fracture of the patella depend largely upon the manner in which the accident occurs. When a man's knee-cap is smashed by being kicked, or by his falling upon it, although the local injury may be very severe, the fragments do not tend to be torn apart, and many of the distressing after consequences are escaped; but when, as is usually the case, the bone is torn across by the violent action of the muscles, as when a man endeavours to save himself when slipping, considerable separation of the fragments is apt to occur, and unless they can be brought together bone to bone true union does not take place, and much weakness of limb is apt to result. Such cases have till recent years been almost universally treated by prolonged rest, the limb being kept straight by a long back splint, the ingenuity of the surgeon having been chiefly expended in devising various means for bringing the fragments together and holding them there. With the exception, however, of Malgaique's hooks—which, although somewhat savage looking, would have answered fairly well if antiseptics had been current in those days—the results of none of these contrivances were very satisfactory, and of late years many new plans have been introduced with the object of introducing greater certainty into the treatment of this injury.

Two points especially have been much practically proved; first, that the maintenance of the separation of the fragments is not altogether due to muscular action, but is largely the result of effusion within the joint; and, second, that unless actual bone-to-bone apposition can be obtained bony union will not take place. As is only natural, these facts, together with the growing safety of and growing confidence in aseptic surgery, have led to a largely extended use of operative methods of treatment. According to one method, a silver wire is passed subcutaneously, so as to embrace the fragments of bone, passing in front of them and behind them, and holding them firmly together; in others the wires are passed through the bone without interfering with the surface of the joint; in another the wire is made to encircle the bone, on the flat, if one may so express it; while again, in another, long needles are passed through the tendon above and below the bony fragments, which, by aid of the projecting ends of the needles, are held in position without the joint itself being opened. The results of most of these measures are admirable, but it has to be borne in mind that none are absolutely devoid of danger, and a strong feeling has lately grown up among many surgeons in favour of the completely open operation. If, it is said, risks have to be run let us get the full benefit which operation can give, one of which is the making certain that the bones are actually in contact with no fibrous tissue intervening, and this can only be obtained by the open operation.

We earnestly hope that none of these problems will have to be discussed in regard to the Prince of Wales, and that rest and fixation will do all that is necessary. It has, however, to be remembered that prolonged rest and prolonged fixation are often in themselves injurious, and that while the hospital patient sometimes asks for operation because it restores to him his power of earning wages, even the most highly placed among us may prefer whatever may best promise a quick restoration of the power of taking exercise.

